# Targeting c-fms kinase attenuates chronic aristolochic acid nephropathy in mice

**DOI:** 10.18632/oncotarget.7460

**Published:** 2016-02-17

**Authors:** Xiao Y. Dai, Xiao R. Huang, Li Zhou, Lin Zhang, Ping Fu, Carl Manthey, David J. Nikolic-Paterson, Hui Y. Lan

**Affiliations:** ^1^ Department of Medicine & Therapeutics, Li Ka Shing Institute of Health Sciences, and Shenzhen Research Institute, The Chinese University of Hong Kong, Hong Kong, China; ^2^ Division of Nephrology, Mianyang Central Hospital, Mianyang, China; ^3^ Division of Nephrology, West China Hospital of Sichuan University, Chengdu, China; ^4^ Janssen Research & Development, LLC, Radnor, PA, USA; ^5^ Department of Nephrology, Monash Health and Monash University Department of Medicine, Clayton, VIC, Australia

**Keywords:** aristolochic acid nephropathy, fms-I, macrophages, inflammation, fibrosis, Pathology Section

## Abstract

Aristolochic acid nephropathy (AAN) is a progressive kidney disease caused by some Chinese herbal medicines, but treatment remains ineffective. Macrophage accumulation is an early feature in human and experimental AAN; however, the role of macrophages in chronic AAN is unknown. We report here that targeting macrophages with fms-I, a selective inhibitor of the tyrosine kinase activity of the macrophage colony-stimulating factor receptor, suppressed disease progression in a mouse model of chronic AAN. Treatment with fms-I (10mg/kg/BID) from day 0 to 28 (prevention study) or from day 14 to 28 (intervention study) substantially inhibited macrophage accumulation and significantly improved renal dysfunction including a reduction in proteinuria and tubular damage. Progressive interstitial fibrosis (myofibroblast accumulation and collagen deposition) and renal inflammation (increased expression of MCP-1, MIF, and TNF-α) were also attenuated by fms-I treatment. These protective effects involved inhibition of TGF-β/Smad3 and NF-kB signaling. In conclusion, the present study establishes that macrophages are key inflammatory cells that exacerbates progressive tubulointerstitial damage in chronic AAN via mechanisms associated with TGF-β/Smad3-mediated renal fibrosis and NF-κB-driven renal inflammation. Targeting macrophages via a c-fms kinase inhibitor may represent a novel therapy for chronic AAN.

## INTRODUCTION

Chronic aristolochic acid nephropathy (AAN) is a progressive form of kidney disease that commonly leads to end-stage of renal failure and urological cancers with no effective treatment. Clinically, patients with AAN exhibit a rapid loss of renal function with tubular damage and massive interstitial nephritis [1-6]. A similar disease course occurs upon administration of aristolochic acid to rats and mice, providing useful models to examine pathogenic mechanisms of interstitial fibrosis and for pre-clinical testing of new therapeutics in AAN [7-11]. Pathologically, chronic human and experimental AAN is characterized by rapid and extensive tubulointerstitial fibrosis with atrophy and loss of the tubules [5, 9, 11, 12].

Interstitial leukocyte accumulation and, in particular, macrophage infiltration is a prominent feature in both human and experimental AAN prior to the development of acellular interstitial scarring [7, 9, 11, 12]; although the functional role of this infiltrate remains unclear. Interstitial macrophage accumulation correlates with interstitial fibrosis across a diverse range of kidney diseases [13-15], and macrophage depletion studies have identified a pathologic role for macrophages in various animal models of renal inflammation and fibrosis [16-19]. However, macrophages are a heterogeneous population and some subsets have been shown to promote repair of damaged tubules albeit after cessation of the underlying cause of injury [20-22]. Therefore, we sought to define the function of macrophages in the development and progression of experimental AAN.

Macrophage-colony stimulating factor (M-CSF) signals through its receptor tyrosine kinase c-*fms* to promote macrophage proliferation, differentiation, and survival [23]. Expression of c-*fms* is restricted to the monocyte/macrophage lineage. Blockade of c-*fms* using neutralizing antibodies or small molecule inhibitors of c-fms kinase activity are effective strategies to selectively deplete macrophages from the diseased kidney [18, 20-22, 24]. Thus, in the current study, we used an inhibitor of the tyrosine kinase activity of c-*fms* to investigate the functional role of macrophages in a mouse model of chronic AAN. The results show that reversal of the macrophage infiltrate halted the progression of established AAN.

## RESULTS

### Chronic aristolochic acid administration induces severe renal injury

**Figure 1:**
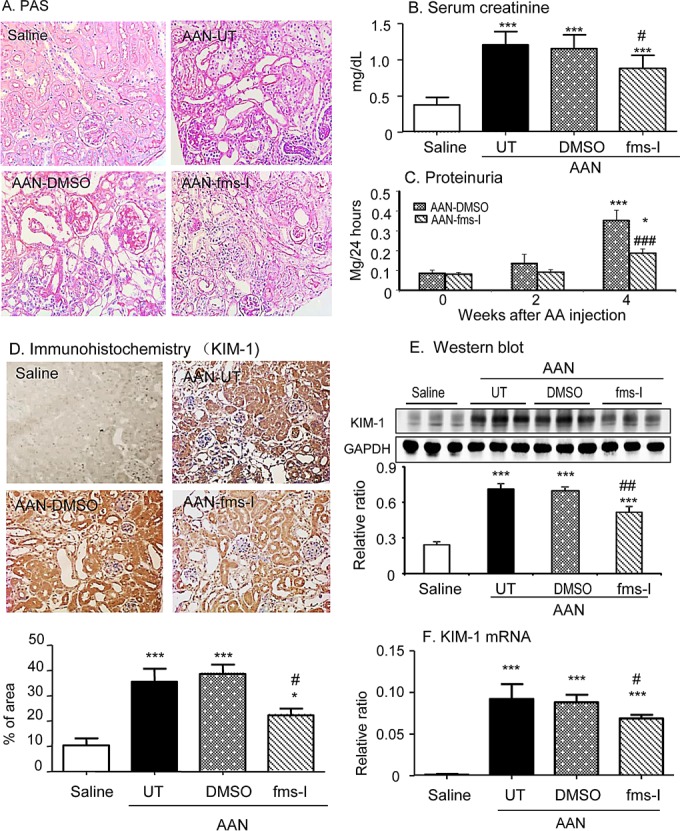
Treatment with fms-I (day 0 to 28) in the prevention study inhibited histological and functional injury in chronic AAN **A.** PAS-staining. **B.** Serum levels of creatinine. **C.** Proteinuria. **D.** KIM-1 expression by immunohistochemical staining. **E.** Western blot analysis of KIM-1 protein expression. **F.** KIM-1 mRNA expression by real-time PCR. Results show that compared to untreated (UT) or vehicle (DMSO) treatment, fms-I treatment significantly inhibited renal histological and functional injury in chronic AAN. Data are expressed as mean ± SE for groups of 6 mice. *p < 0.05, **p < 0.01, ***p < 0.001 compared with saline control. ^#^*p* < 0.05, ^##^*p* < 0.01, ^###^*p* < 0.001 compared with untreated or vehicle (DMSO) treated chronic AAN. Magnification: x200.

Administration of aristolochic acid (AA) to untreated and vehicle treated mice resulted in typical features of chronic AAN; marked tubular damage with atrophy, dilatation and bared tubular basement membrane accompanied by severe tubulointerstitial fibrosis (Figure 1A). Glomeruli retained a relatively normal appearance. Both renal impairment, based on elevated serum creatinine, and proteinuria were evident on day 28 (Figure 1B and 1C). Consistent with the severity of tubular damage seen on PAS stained sections, the biomarker of tubular damage, KIM-1, was markedly increased on day 28 in untreated and vehicle treated AAN (Figure 1D-1F).

**Figure 2:**
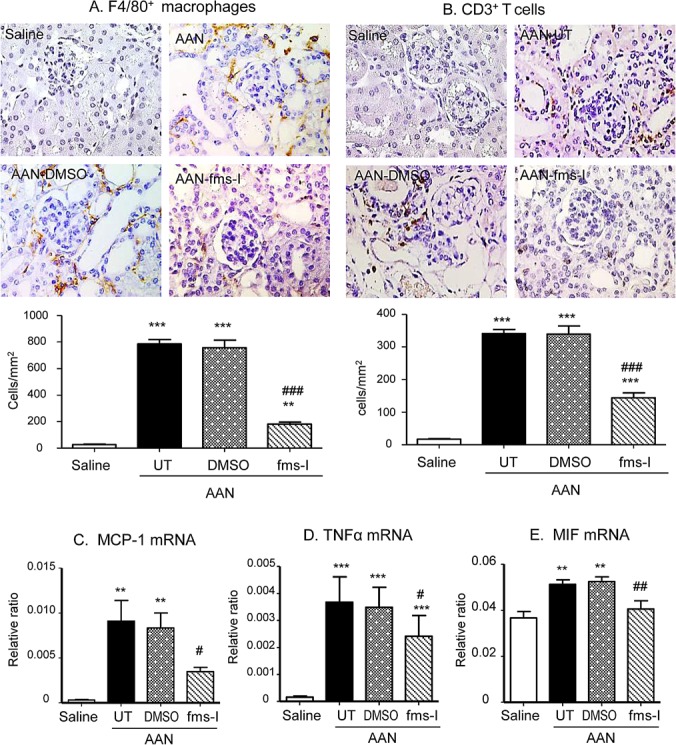
Treatment with fms-I (day 0 to 28) in the prevention study inhibited macrophage accumulation and kidney inflammation in chronic AAN Immunohistochemical staining and quantification of: **A.** F4/80^+^ macrophages, and **B.** CD3^+^ T cells. **C.**-**E.** Real-time PCR analysis of MCP-1, TNF-α and MIF mRNA levels. Results show that compared to untreated (UT) or vehicle (DMSO) treatment, fms-I treatment markedly reduced F4/80^+^ macrophage accumulation in chronic AAN. Fms-1 treatment also reduced CD3^+^ T cell infiltration and upregulation of pro-inflammatory cytokines. Data are expressed as mean ± SE for groups of 6 mice. **p < 0.01, ***p < 0.001 compared with saline control. ^#^*p* < 0.05, ^##^*p* < 0.01, ^###^*p* < 0.001 compared with untreated or vehicle (DMSO) treated chronic AAN. Magnification: x400.

**Figure 3:**
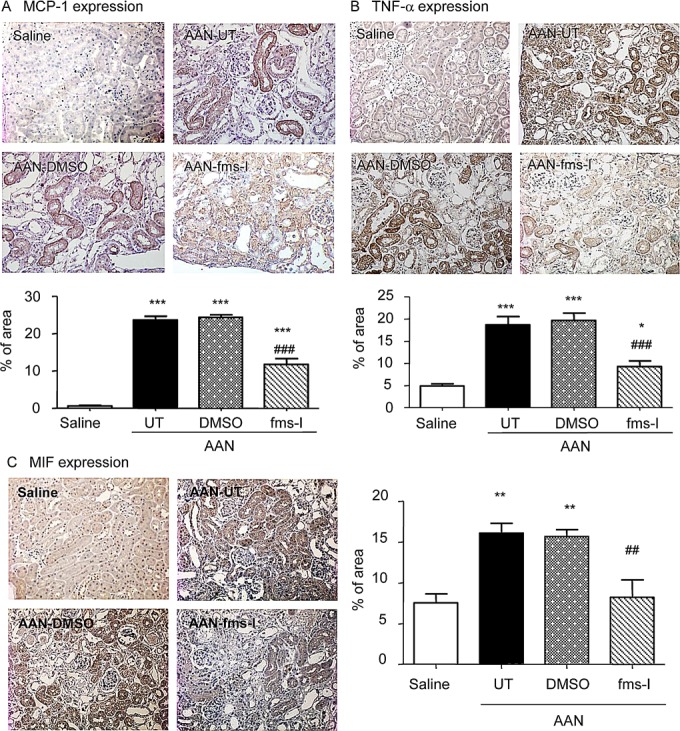
Treatment with fms-I (day 0 to 28) in the prevention study inhibited up-regulation of pro-inflammatory cytokines in chronic AAN Immunohistochemical staining is shown for: **A.** MCP-1, **B.** TNFα, and **C.** MIF expression. Results show that compared to untreated (UT) or vehicle (DMSO) treatment, fms-I treatment substantially inhibited the upregulation of pro-inflammatory cytokines. Data are expressed as mean ± SE for groups of 6 mice. *p < 0.05, **p < 0.01, ***p < 0.001 compared with saline control. ^##^*p* < 0.01, ^###^*p* < 0.001 compared with untreated or vehicle (DMSO) treated chronic AAN. Magnification: x200.

**Figure 4:**
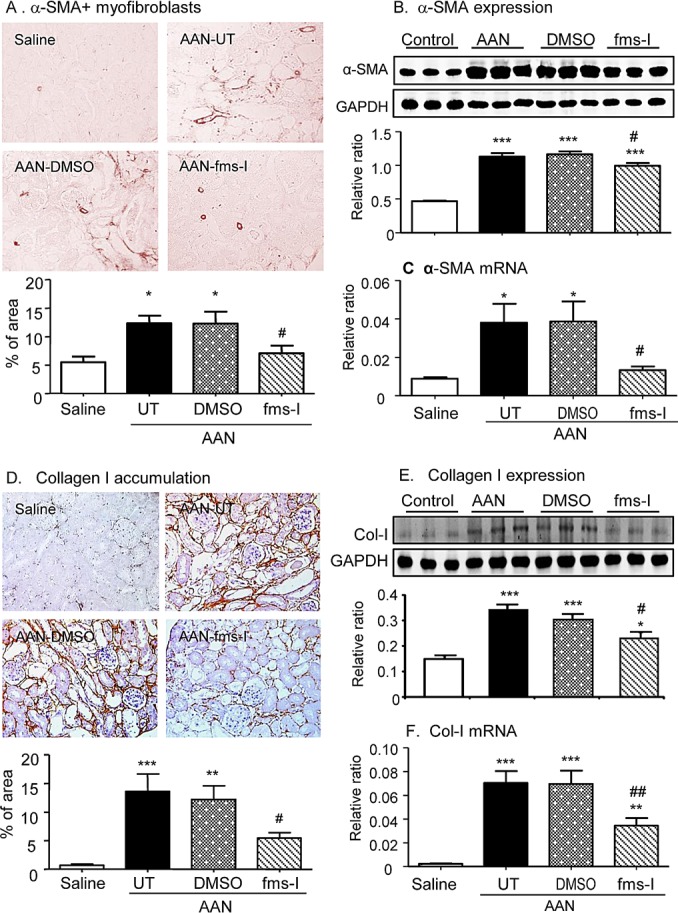
Treatment with fms-I (day 0 to 28) in the prevention study inhibited renal fibrosis in chronic AAN **A.** Immunohistochemical staining for α-SMA^+^ myofibroblasts. **B.** Western blot analysis of α-SMA protein levels. **C.** Real-time PCR analysis of α-SMA mRNA levels. **D.** Immunohistochemical staining for collagen I. **E.** Western blot analysis of collagen I protein levels. **F.** Real-time PCR analysis of collagen I mRNA levels. Results show that compared to untreated (UT) or vehicle (DMSO) treatment, fms-I treatment substantially reduced the accumulation of α-SMA^+^ myofibroblasts and the deposition of collagen I. Data are expressed as mean ± SE for groups of 6 mice. *p < 0.05, **p < 0.01, ***p < 0.001 compared with saline control. ^#^*p* < 0.05, ^##^*p* < 0.01 compared with untreated or vehicle (DMSO) treated chronic AAN. Magnification: x200.

A prominent interstitial accumulation of F4/80^+^ macrophages was seen on day 28 in both untreated and vehicle treated mice. A significant though less prominent T cell infiltrate was also evident (Figure 2A and 2B). These infiltrates were accompanied by up-regulation of the pro-inflammatory and chemotactic molecules monocyte chemoattractant protein-1 (MCP-1), macrophage migration inhibitory factor (MIF) and TNF-α at the mRNA level (Figure 2C-2E). Immunohistochemistry staining identified tubular epithelial cells as the major site of production of these pro-inflammatory molecules (Figure 3). Mice developed significant interstitial fibrosis on day 28 of AAN as evident by the accumulation of α-SMA+ myofibroblasts and interstitial deposition of collagen I (Figure 4A-4F).

### fms-I treatment suppresses the development of AAN

Based upon a pilot study (Suppl Fig S1), we chose a dose of 10mg/kg fms-I in the prevention study of AAN. Treatment with fms-I from day 0 to 28 reduced the severity of tubular damage based upon PAS histology and KIM-1 expression (Figure 1A and 1D), and reduced the severity of renal impairment and proteinuria (Figure 1B and 1C). This was accompanied by a 75% reduction in the macrophage infiltrate (Figure 2A). The up-regulation of pro-inflammatory molecules and the T cell infiltrate was also reduced by fms-I treatment (Figure 2B-2E and Figure 3). In addition, fms-I treatment reduced the accumulation of α-SMA+ myofibroblasts and inhibited the deposition of interstitial collagen I by over 50% (Figure 4).

### fms-I treatment halts the progression of established AAN

**Figure 5:**
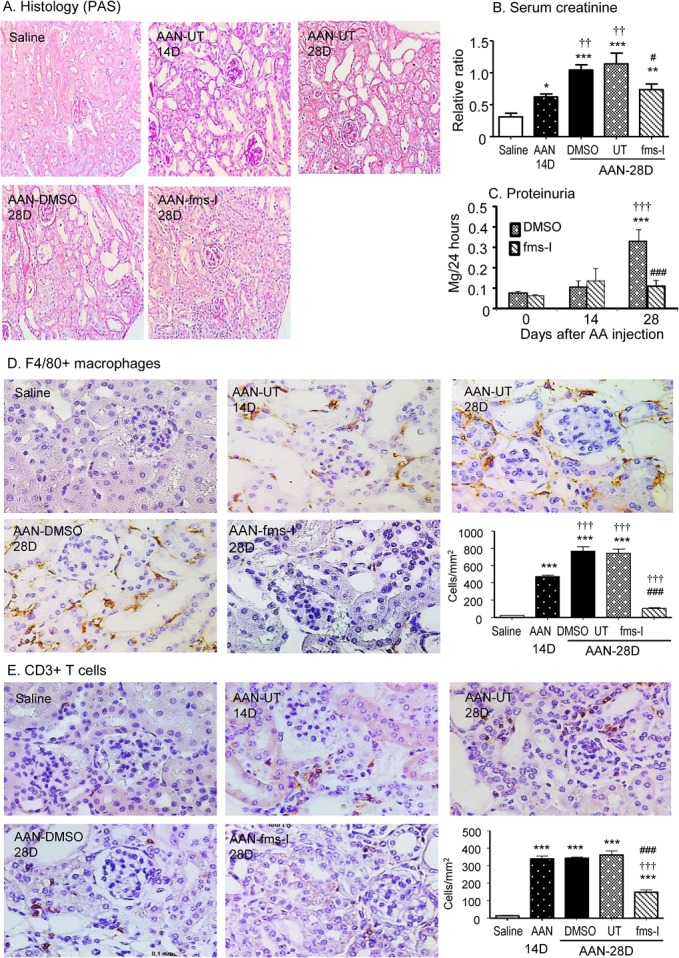
Treatment with fms-I (day 14 to 28) in the intervention study reverses macrophage accumulation and inhibits renal injury and inflammation in established AAN **A.** PAS-staining. **B.** Serum levels of creatinine. **C.** Proteinuria. **D.**-**E.** Immunohistochemical staining for F4/80+ macrophages and CD3 + T cells. Results show that compared to untreated (UT) or vehicle (DMSO) treatment, fms-I treatment in established AAN halted progressive loss of renal function and prevented proteinuria in associated with reversal of macrophage and T cell accumulation and a reduction in pro-inflammatory cytokine gene expression. Data are expressed as mean ± SE for groups of 6 mice. *p < 0.05, **p < 0.01, ***p < 0.001 compared with saline control. ^#^*p* < 0.05, ^###^*p* < 0.001 compared with day 28 chronic AAN with vehicle (DMSO) treatment or untreated (UT).†*p* < 0.05, ††*p* < 0.01, †††*p* < 0.001 compared with day 14 disease before fms-I treatment. Magnification: x200 (A).

**Figure 6:**
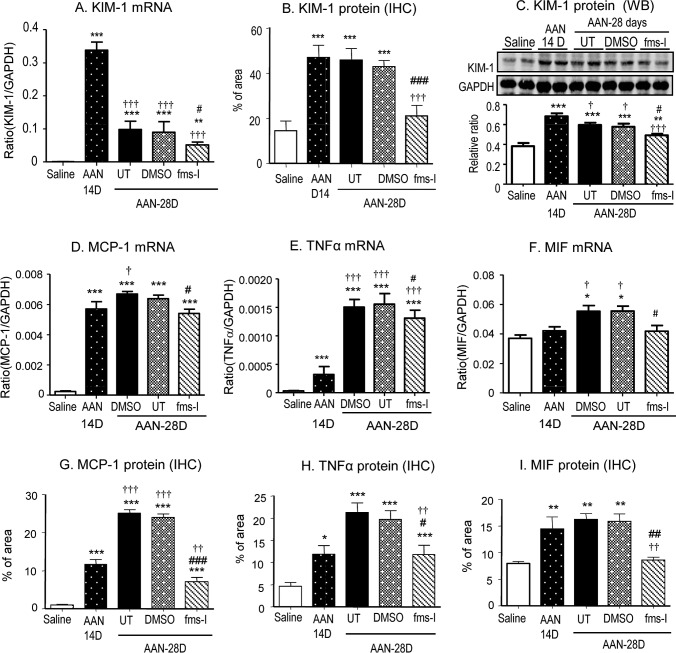
Treatment with fms-I (day 14 to 28) in the intervention study inhibits tubular damage and renal inflammation in established AAN **A.**-**C.** Expression of the biomarker of tubular damage, KIM-1, was assessed by real-time PCR **A.**, immunohistochemistry **B.**, and Western blotting **C.**. **D.**-**F.** Real-time PCR analysis of MCP-1, TNF-α and MIF mRNA levels. **G.**-**I.** Quantification of immunohistochemical staining for MCP-1 **G.**, TNFα (H), and MIF(I) expression. Data are expressed as mean ± SE for groups of 6 mice. *p < 0.05, **p < 0.01, ***p < 0.001 compared with saline control mice. ^#^*p* < 0.05, ^##^*p* < 0.01, ^###^*p* < 0.001 compared with untreated or vehicle (DMSO) treated chronic AAN; ^†^*p* < 0.05, ^††^*p* < 0.01, ^†††^*p* < 0.001 compared with day 14 disease before fms-I treatment. Magnification: x200.

**Figure 7:**
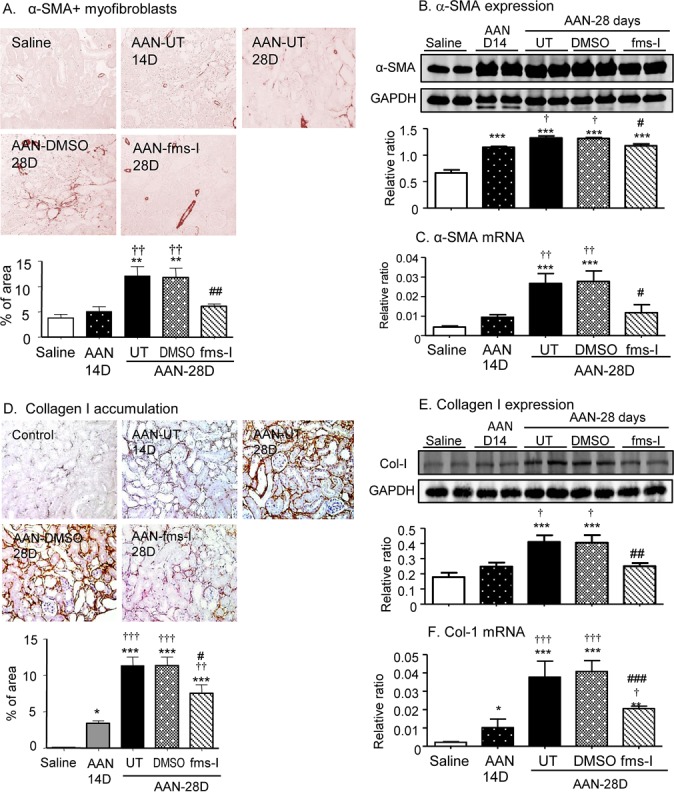
Treatment with fms-I (day 14 to 28) in the intervention study inhibits renal fibrosis in established chronic AAN **A.** Immunohistochemical staining for α-SMA^+^ myofibroblasts. **B.** Western blot analysis of α-SMA protein levels. **C.** Real-time PCR analysis of α-SMA mRNA levels. **D.** Immunohistochemical staining for collagen I. **E.** Western blot analysis of collagen I protein levels. F. Real-time PCR analysis of collagen I mRNA levels. Data are expressed as mean ± SE for groups of 6 mice. *p < 0.05, **p < 0.01, ***p < 0.001 compared with saline control. ^#^*p* < 0.05, ^##^*p* < 0.01, ^###^*p* < 0.001 compared with day 28 chronic AAN with vehicle (DMSO) treatment or untreated (UT).†*p* < 0.05, ††*p* < 0.01, †††*p* < 0.001 compared with day 14 disease before fms-I treatment. Magnification: x200

To determine whether c-fms inhibition has therapeutic potential for chronic AAN, we performed an intervention study with fms-I *versus* vehicle treatment over days 14 to 28 and compared this to untreated (UT) AAN. Mice exhibited severe tubular damage on day 14 of AAN as shown by PAS staining and a peak of KIM-1 expression (Figures 5A and 6A-6C). A significant increase in serum creatinine was also evident on day 14 of untreated AAN, together with a substantial infiltrate of F4/80+ macrophages and CD3+ T cells (Figure 5B-5E) and up-regulation of MCP-1, TNFα, and MIF in both mRNA and protein levels (Figure 6D-6I). In addition, mild interstitial fibrosis was seen on day 14 of AAN (Figure 7). Treatment with fms-I over days 14 to 28 halted the progression of established AAN as shown by preventing a further loss of renal function, preventing the development of proteinuria and less severe tubular damage based on PAS staining and KIM-1 expression (Figures 5A-5C and 6A-6C). This halting of disease progression was associated with a reversal of the F4/80+ macrophage infiltrate, reduced T cell infiltration and reduced expression levels of pro-inflammatory molecules MCP-1, MIF and TNF-α (Figures 5D-5E and 6D-6I). In addition, intervention with fms-I treatment substantially reduced the increase in α-SMA+ myofibroblast accumulation and collagen I deposition seen over days 14 to 28 in vehicle and untreated AAN (Figure 7).

### Analysis of pro-inflammatory and pro-fibrotic signaling pathways in AAN

**Figure 8:**
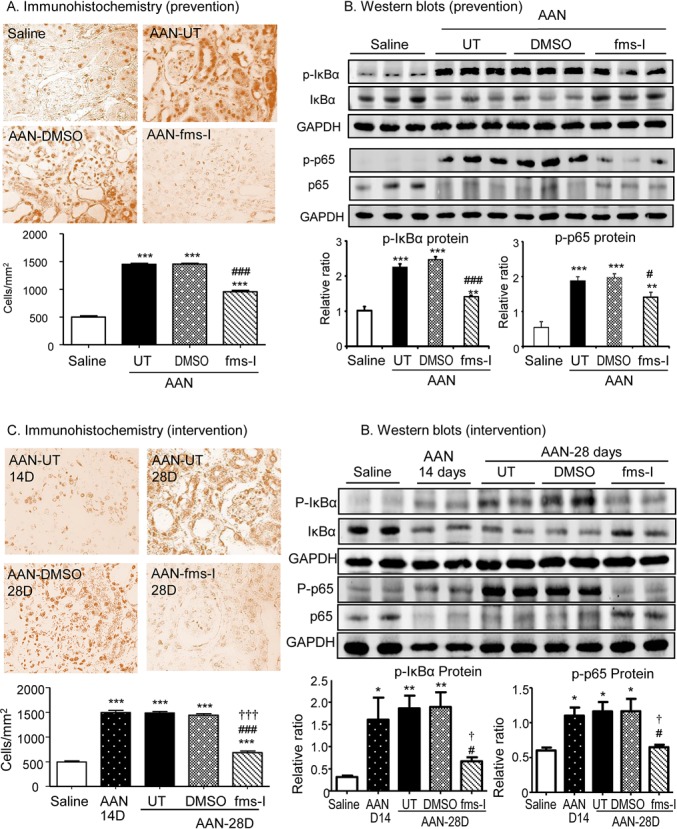
Treatment with fms-I inhibits activation of NF-kB signaling in chronic AAN **A.** and **B.** Prevention study (day 0-28). **C.** and **D.** Interventional study (day 14-28). Results show that treatment with a fms-inhibitor blocks phospho-NF-kB/p65 nuclear translocation and phospho-IkBα and phospho-p65 protein degradation in both studies. Data are expressed as mean ± SE for groups of 6 mice. *p < 0.05, **p < 0.01, ***p < 0.001 compared with saline control. ^#^*p* < 0.05, ^##^*p* < 0.01, ^###^*p* < 0.001 compared with chronic AAN treated with or without vehicle treatment. ^†^*p* < 0.05, ^†††^*p* < 0.001 compared with day 14 disease before fms-I treatment. Magnification: x400 (A, C)..

We next examined the potential signaling mechanisms through which macrophages promote inflammation and fibrosis in the AAN model. NF-kB is a transcription factor that drives gene transcription of many pro-inflammatory molecules, including MCP-1 and TNF-α [25]. A substantial increase in NF-kB activation was evident in both tubular and interstitial cells based on immunostaining for phospho-p65 nuclear translocation and western blot analysis of phosphorylation of the p65 subunit and the loss of the inhibitory IkBα subunit (Figure 8). Treatment with fms-I in both the prevention and intervention studies substantially reduced NF-kB activation on day 28 of AAN (Figure 8).

**Figure 9:**
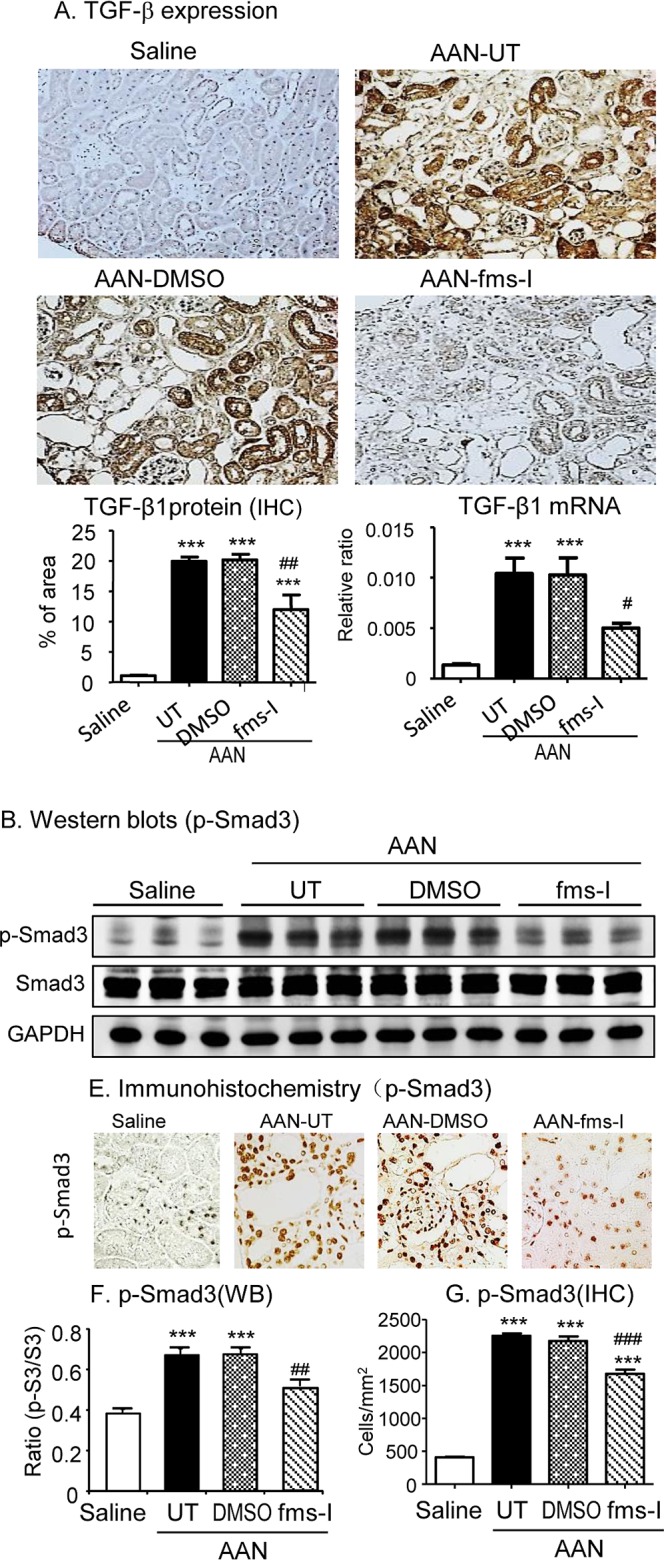
Treatment with fms-I (day 0-28) inhibits TGF-β/Smad signaling in chronic AAN **A.** Immunohistochemical staining and real-time PCR for expression of TGF-β1. **B.** Western blot analysis and immunohistochemistry for activation of Smad3 signaling illustrated by phospho-Smad3 nuclear translocation and protein phosphorylation. Data are expressed as mean ± SE for groups of 6 mice. ***p < 0.001 compared with saline control. ^#^*p* < 0.05, ^##^*p* < 0.01, ^###^*p* < 0.001 compared with chronic AAN treated with or without vehicle treatment. Magnification: x200 (A) or x400 (B).

**Figure 10:**
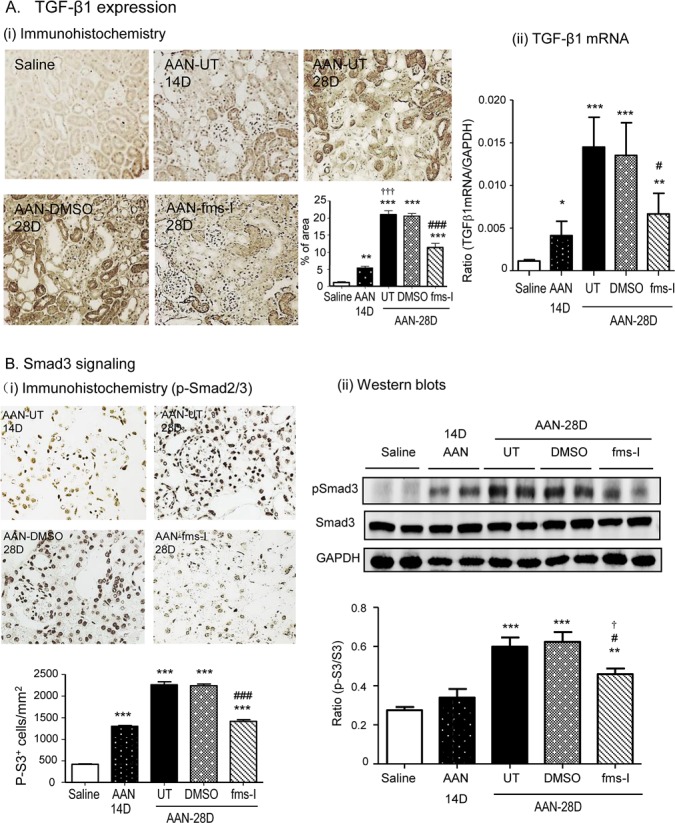
Treatment with fms-I (day 14-28) inhibits TGF-β/Smad signaling in established chronic AAN **A.** Immunohistochemical staining and real-time PCR for expression of TGF-β1. **B.** Immunohistochemistry and Western blot analysis of Smad3 signaling illustrated by phospho-Smad3 nuclear translocation and protein phosphorylation. Data are expressed as mean ± SE for groups of 6 mice. *p < 0.05, **p < 0.01, ***p < 0.001 compared with saline control. ^#^*p* < 0.05, ^###^*p* < 0.001 compared with chronic AAN treated with or without vehicle treatment. ^†^*p* < 0.05 compared with day 14 disease before fms-I treatment. Magnification: x200 (A) or x400 (B).

The TGF-β/Smad3 signalling pathway plays a key role in the development of renal fibrosis [26]. Analysis of whole kidney tissue identified a substantial increase in TGF-β1 mRNA levels on day 28 of AAN and increased TGF-β1 production was predominantly localized to tubular epithelial cells (Figure 9A). Western blotting identified a significant increase in the level of phosphorylated Smad3 in AAN, while immunostaining localized p-Smad3 to nuclei of tubular epithelial and interstitial cells (Figure 9B). Prevention treatment with fms-I reduced both TGF-β1 expression levels and Smad3 phosphorylation on day 28 AAN (Figure 9), while intervention treatment with fms-I largely halted the increase in TGF-β1 expression and Smad3 phosphorylation (Figure 10).

## DISCUSSION

The prominent macrophage accumulation seen in the current study of experimental AAN is consistent with studies of human AAN in which macrophage infiltration occurs prior to the development of acellular interstitial fibrosis [3, 5, 6, 27], and with previous studies of experimental AAN [7-9, 11]. Interstitial macrophage accumulation in this model was associated with up-regulation of tubular expression of the chemotactic molecules, MCP-1 and MIF, which have been shown to promote monocyte recruitment in a several models of kidney disease [28-31].

Aristolochic acid induced tubular damage is the primary insult in AAN. Interestingly, a reduction in tubular damage was evident with fms-I mediated macrophage depletion demonstrating that macrophages contribute to tubulointerstitial damage in this model by exacerbating tubular damage. Aristolochic acid causes DNA damage in tubular epithelial cells resulting in the production of reactive oxygen species, secretion of chemokines, cell cycle arrest and apoptosis [32, 33]. The production of monocyte chemotactic molecules MCP-1 and MIF by tubular epithelial cells in response to aristolochic acid toxicity is likely to account for the recruitment of blood monocytes into the injured tubulointerstitium in AAN. Our findings indicate that macrophages entering the injured tubulointerstitium are activated to exacerbate tubular damage which is consistent with other models of renal injury in which macrophages cause tubular damage and apoptosis [34, 35].

Tubular epithelial cells are major drivers of interstitial fibrosis in the kidney. Tubules are the chief source of TGF-β1 in interstitial fibrosis [11, 36]. The marked reduction in tubular TGF-β1 expression seen with fms-1 treatment indicates that macrophages act on tubular epithelial cells to increase TGF-β1 production, possibly through secretion of pro-inflammatory factors such as IL-1 and TNF-α [37, 38]. Tubular cells were also identified as major sites of TGF-/Smad signalling based on p-Smad3 immunohistochemistry. The reduction in Smad3 activation in tubular cells with depletion of kidney macrophages implies that either macrophages produce active TGF-β1, or active extracellular latent TGF-β1, to induce TGF-β/Smad3 signalling in tubular cells. Macrophages can liberate latent TGF-β1 from extracellular matrix through secreted proteases, and they can activate latent TGF-β1 *via* several mechanisms including cell surface integrins and secretion of MMP-9 and thrombospondin [39]. These findings are consistent with a role for macrophage in renal fibrosis [15], and the demonstration that reversal of macrophage accumulation in the chronic phase of crescentic glomerulonephritis significantly reduced both glomerular and interstitial fibrosis [17].

A second interesting finding was that macrophages were required for the full pro-inflammatory response of tubular epithelial cells in the AAN model as shown by activation of the transcription factor NF-kB. This finding is consistent with co-culture experiments in which monocytes were shown to induce NF-kB activation of tubular epithelial cells [40]. In addition, macrophage products such as IL-1 and TNF-α are potent inducers of NF-kB activation in tubular epithelial cells [41]. This suppression of NF-kB activation may explain the partial reduction in tubular production of the pro-inflammatory molecules MCP-1, MIF and TNF-α seen with fms-I treatment.

Fms-I is a selective c-fms kinase inhibitor. We have shown selective inhibition of macrophage accumulation in several models of renal injury, including unilateral ureteric obstruction, crescentic glomerulonephritis and renal allograft rejection [16-19]. This operates *via* several mechanisms including; inhibition of local macrophage proliferation and, at high doses (30mg/kg) *via* suppression of blood monocyte counts [16, 18]. In the present study, we identified a 75% reduction in macrophage accumulation in AAN, which was also effective when commenced after macrophage accumulation was established. The reduction seen in T cell accumulation in AAN is most likely due to reduced tubular damage and T cell chemokine production rather than an off-target effect since we have previously shown that fms-I, even at 30mg/kg, did not alter the recruitment of T cells in renal allograft rejection and high concentrations of fms-I failed to inhibit T cell proliferation *in vitro* [19]. However, as in all studies of this type, we cannot exclude the possibility that fms-I exerted a protective effect in tubular epithelial cells through an off-target action.

In conclusion, we have established an important role for macrophages in the development and progression of AAN through exacerbation of tubular epithelial cell damage and enhancing the tubular pro-inflammatory and pro-fibrotic responses. In particular, the ability of fms-I treatment to suppress the progression of established disease in experimental AAN identifies c-fms kinase inhibitors as a potential new therapy for chronic AAN.

## MATERIALS AND METHODS

### Animal model of chronic AAN

A chronic AAN model was induced in C57/BL6 mice (male, aged 8 weeks) by intraperitoneal injection of 5mg/kg aristolochic acid (Sigma, St. Louis, MO) every other day for 4 weeks as described previously [11]. Twenty-four hour urine samples were collected in metabolic cages before and every 2 weeks after induction of AAN to assay proteinuria. Groups of 6 mice were killed at 4 weeks after the initial AA injection. In addition, a group of 6 normal C57/BL6 mice received saline injection was used as normal age-matched control.

A pilot study was performed in the AAN model using 5, 10 and 20mg/kg fms-I delivered by twice daily intraperitoneal injection in 0.1% DMSO vehicle. Based upon the lowest dose for maximal reduction in macrophage accumulation, the main study used 10mg/kg/bid fms-I (Suppl Fig S1). In the prevention study, groups of 6 AAN mice were untreated or given fms-I or vehicle twice daily from day 0 until being killed on day 28. In the intervention study, groups of 6 AAN mice were given fms-I or vehicle treatment from day 14 until being killed on day 28. In addition, groups of untreated AAN mice were killed on day 14 or 28. Kidney function was assessed from a terminal blood sample. Twenty-four hour urine samples were collected in metabolic cages before and every 2 weeks after induction of AAN to assay proteinuria. The experimental procedures were approved by the Institutional Animal Experimentation Ethics Committee (Permit No. 12-352).

### c-fms kinase inhibitor

fms-I (4-cyano-1H-imidazole-2-carboxylic acid {2-cyclohex-1-enyl-4-[1-(2-methanesulfonyl-ethyl)-piperidin-4-yl]-phenyl}-amide) is a selective inhibitor of the tyrosine kinase activity of c-fms synthesized by Johnson & Johnson Pharmaceutical Research and Development [18]. The compound was dissolved in DMSO for use.

### Renal function and proteinuria

Urine protein levels were measured using the Quick Start Bradford Dye Reagent (BioRAD). Serum creatinine was measured using the Enzymatic Creatinine Liquid Color Reagent (Stanbio Laboratory, Boerne, TX), according to the manufacturer's instructions.

### Histology and immunohistochemistry

Histological assessment was performed in 4μm paraffin sections of renal tissues fixed in methyl Carnoy's solution and stained with periodic acid-Schiff (PAS) reagent. Immunohistochemistry was performed on formalin-fixed paraffin sections using a microwave-based antigen retrieval technique [42]. Primary antibodies used in the study were as followed: collagen I (Southern Technology, Birmingham, AL), α-SMA (Sigma, St. Louis, MO), TNFα, MCP-1, MIF, TGF-β1, phospho-Smad2/3 (Santa Cruz Biotechnology, Santa Cruz, CA), phospho-NFκB/p65, CD3, KIM-1 (Abcam, Cambridge, MA), and F4/80 (Serotec, Oxford, UK). After being immunostained with the secondary antibodies, sections were developed with diaminobenzidine to produce a brown product. All slides were counterstained with hematoxylin except for phospho-Smad2/3 and phospho-NFκB/p65 immunodetection. The percentage of positive staining for KIM-1, collagen I, αSMA, TNFα, MCP-1, MIF, TGF-β1 was measured by using a quantitative image-analysis system (Image-Pro Plus 6.5, Media Cybernetics, Silver Spring, MD), omitting staining of large arteries for collagen I and α-SMA, while the number of cells positively stained for phospho-p65, phospho-Smad2/3, CD3 or F4/80 were counted in the tubulointerstitium in high-power fields (×40) by means of a 0.0625-mm^2^ graticule fitted in the eyepiece of the microscope and expressed as cells per square millimeter.

### Real-time PCR

RNA was collected from renal tissues and purified by an RNeasy kit according to the manufacturer's instructions (Qiagen, Valencia, CA), and real-time PCR was performed with Sybergreen on an Opticon real-time PCR machine (MJ Research,Waltham, MA) as previously described [43, 44]. Primers used for detection of mRNA expression of KIM-1, MIF, collagen I, α-SMA, TGF-β1, MCP-1, TNFα, and GAPDH were described previously [27, 45]. GAPDH was used as an internal standard. The ratio for the mRNA was examined against GAPDH and was expressed as mean ± SE.

### Western blot analysis

Protein from renal tissues were extracted with RIPA lysis buffer and analyzed by Western blotting as previously described [46, 47]. Briefly, after protein was transferred onto a nitrocellulose membrane, the membrane was incubated at 4°C overnight with primary antibodies against phospho-p65 (Ser276), phospho-IκBα (Ser32) and IκBα (Cell Signaling), p65, phospho-Smad2/3 and Smad2/3 (Santa Cruz), collagen I (Southern Biotech), α-SMA (Sigma), KIM-1 (Abcam, Cambridge, MA), GAPDH (Chemicon, Temecula, CA), followed by the LI-COR IRDye 800-labeled secondary antibodies (Rock-land Immunochemicals, Gilbertsville, PA). The signals were detected with an Odyssey Infrared Imaging System (Li-COR Biosciences, Lincoln, NE, USA) and quantified with Image J (National Institutes of Health, Bethesda, MD, USA). The ratio for the protein examined was normalized against GAPDH.

### Statistical analysis

Data obtained from this study are expressed as mean ± SE. Statistical analyses were performed using one-way analysis of variance followed by Newman-Keuls post-test analysis (Prism 5.0 GraphPad Software, San Diego, CA).

## SUPPLEMENTARY FIGURES AND TABLES


