# Supplementary material for: Targeting c-fms kinase attenuates chronic aristolochic acid nephropathy in mice
Source: Oncotarget. 2016 Feb 17;7(10):10841–56. doi: 10.18632/oncotarget.7460 (PMC4905443; doi:10.18632/oncotarget.7460)
Supplement: Supplementary file 1 [file oncotarget-07-10841-s001.pdf]

Targeting c-fms kinase attenuates chronic aristolochic acid nephropathy in mice

Supplementary Material

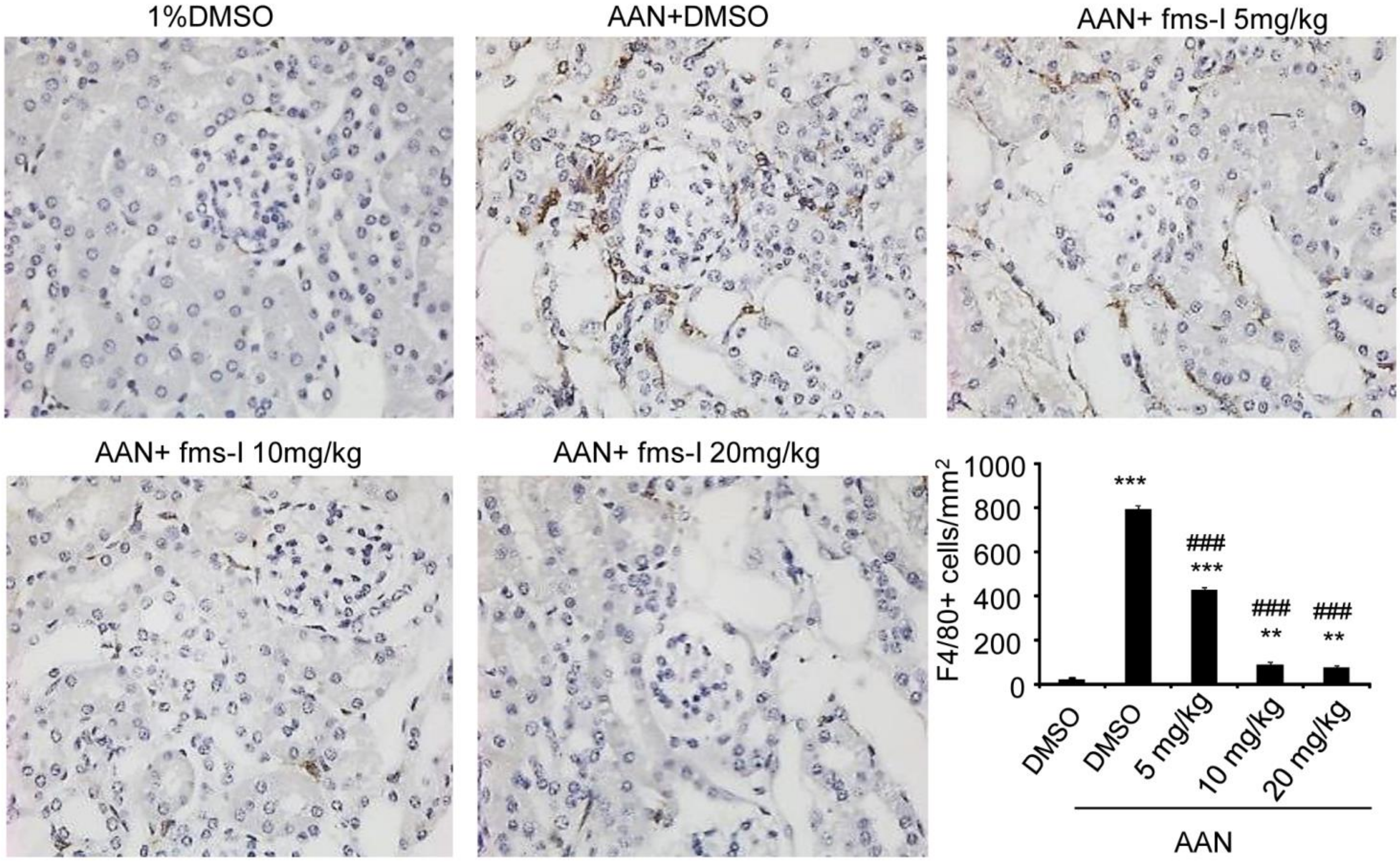

Supplementary figure S1. Pilot study to assess the dose of fms-I required to inhibit macrophage accumulation in the chronic AAN model. Groups of 6 mice with AAN were treated with 5, 10 or 20mg/kg/BID fms-I versus vehicle alone (DMSO) from day 0 to 28. Macrophages in the kidney were detected by immunostaining with the F4/80 antibody. A. Vehicle alone (no aristolochic acid administration) did not cause macrophage infiltration. B. Vehicle treated AAN showed a marked accumulation of F4/80^{+} macrophages. C-E. Increasing fms-I dose resulted in a progressive reduction of macrophage accumulation on day 28 of AAN. G. Graph quantifying macrophage accumulation. Data are expressed as mean ± SE for groups of 6 mice. **P<0.05, ***p<0.001 compared with normal mice treated with vehicle (DMSO); $^{###}$P<0.001 compared to vehicle treated AAN. Magnification: x400.
